# MicroRNA expression profiles and clinicopathological implications in lung adenocarcinoma according to EGFR, KRAS, and ALK status

**DOI:** 10.18632/oncotarget.14298

**Published:** 2016-12-27

**Authors:** Hyojin Kim, Jeong Mi Yang, Yan Jin, Sanghoon Jheon, Kwhanmien Kim, Choon Taek Lee, Jin-Haeng Chung, Jin Ho Paik

**Affiliations:** ^1^ Department of Pathology, Seoul National University Bundang Hospital, Seongnam, Korea; ^2^ Department of Thoracic and Cardiovascular Surgery, Seoul National University Bundang Hospital, Seongnam, Korea; ^3^ Department of Internal Medicine, Seoul National University Bundang Hospital, Seongnam, Korea

**Keywords:** microRNA, lung cancer, adenocarcinoma, miR-342-3p, ALK

## Abstract

Lung adenocarcinoma has distinctive clinicopathological features that are related to specific genetic alterations, including *EGFR* and *KRAS* mutations and *ALK* rearrangement. MicroRNAs are small non-coding RNAs that post-transcriptionally regulate many important biological processes and influence cancer phenotypes. This study retrospectively investigated microRNA expression profiles, and their clinicopathological implications, in lung adenocarcinoma according to genetic status (*EGFR*, *KRAS*, *ALK*, and triple negative). A total of 72 surgically resected lung adenocarcinoma specimens (19 *EGFR-*mutated, 17 *KRAS*-mutated, 16 *ALK*-rearranged, and 20 triple negative cancers) were screened for 23 microRNAs using quantitative real-time reverse transcriptase polymerase chain reaction. We then evaluated the associations between the microRNA expressions and the cancers’ genetic and clinicopathological features. Eight microRNAs were associated with clinicopathological features, such as male sex and ever-smoker status (high miR-373-3p, miR-1343-3p, miR-138-1-3p, and miR-764; low miR-27b-3p) and vascular invasion (high miR-27b-3p; low miR-1343-3p and miR-764). Clustering and discriminant analyses revealed that the microRNA expression patterns in the *ALK* group were different from those in the *EGFR* and *KRAS* groups. Five microRNAs (high miR-1343-3p; low miR-671-3p, miR-103a-3p, let-7e, and miR-342-3p) were especially distinctive in the *ALK* group, compared to the *EGFR* and *KRAS* groups. Moreover, a significant association was observed between *ALK*-rearrangement, decreased miR-342-3p expression, and immunohistochemical loss of E-cadherin. Therefore, microRNA expression profiles appear to have distinctive clinicopathological implications in *ALK-*rearranged lung adenocarcinoma. Furthermore, the association of *ALK* rearrangement, decreased miR-342-3p expression, and E-cadherin loss might indicate that miR-342-3p is involved in the *ALK*-associated phenotypes and epithelial-mesenchymal transition.

## INTRODUCTION

Lung adenocarcinoma is the most common histological type of primary lung cancer [1], and the identification of key oncogenic drivers has created a paradigm shift in our understanding of lung adenocarcinoma biology. Furthermore, lung adenocarcinoma is a heterogeneous tumor with diverse molecular, clinical, and pathological characteristics [2–4], and the mutational status of driver oncogenes can be used to create personalized strategies for treating the advanced disease. Lung adenocarcinomas with activating mutations of the epidermal growth factor receptor gene (*EGFR*) have been reported to respond to *EGFR* tyrosine kinase inhibitors (TKIs) [5–7], and rearrangement of the anaplastic lymphoma kinase gene (*ALK*) is the best predictor of lung adenocarcinoma response to crizotinib (an *ALK* TKI) [8–10].

MicroRNAs are small endogenous non-coding RNAs that can modulate protein expression by regulating translational efficiency or cleavage of the target mRNA [11]. Deregulated microRNA expression has been identified in a variety of human malignancies, which suggests potential oncogenic or tumor-suppressive roles, depending on the cancer cell, tissue, and target gene [12–16]. In this context, microRNA expression profiles are emerging as potentially useful diagnostic and prognostic biomarkers, which can facilitate personalized therapy and disease management [17, 18]. Oncogenic and tumor-suppressive microRNAs are deregulated in the various tumor entities of lung cancer [19–23], and accumulating evidence indicates that dysregulation of specific microRNAs contributes to the development and progression of lung cancer. For example, overexpression of miR-17-92, miR-21, and miR-128 has been observed in lung cancer and has implications in its carcinogenesis [19–21, 24]. Compared to the normal lung, lung adenocarcinoma exhibits dysregulation of several microRNAs, such as let-7a-2, let-7a-3, miR-15b, miR-21, miR-155, and miR-200b, which are associated with the development and progression of cancer (altered tumor suppression) and shorter patient survival (reduced prognosis) [25]. Among these microRNAs, let-7 was the first to be confirmed as having aberrant expression in lung carcinoma [26, 27], and it is now known that the let-7 microRNA family controls the activity of the RAS oncogene [28]. Other microRNAs, such as miR-200b, regulate the chemosensitivity of human lung adenocarcinoma cell lines, mainly through their effects on cell proliferation, cell cycle distribution, and apoptosis [29].

Few studies have investigated microRNA expressions and genetic profiles in lung cancer, although recent studies have demonstrated that *EGFR* may be a functional target of microRNAs [24, 30]. In this context, miR-128b is frequently deleted in lung cancers and directly downregulates *EGFR*, but is not associated with *EGFR* mutational status and prognosis [24]. Another study demonstrated that miR-96 decreased the phosphorylation of *ALK* target proteins, and reduced the proliferation, colony formation, and migration of ALK-expressing cancer cells, which suggests that miR-96 may be involved in the aberrant expression of ALK in cancer cells [31]. However, little is known regarding any differences in microRNA expressions in lung adenocarcinomas with different oncogene mutational statuses. Therefore, the present study aimed to 1) investigate the microRNA expression profiles in surgically resected lung adenocarcinomas with different oncogenic mutations, 2) analyze their clinicopathological implications, and 3) evaluate any associations between the mutation-related phenotypes and microRNAs. This information may help improve our understanding of tumor behavior according to mutational status, and might help identify therapeutic strategies for lung adenocarcinoma that are based on the use of specific microRNAs.

## RESULTS

### Clinicopathological characteristics of patients with lung adenocarcinomas

The clinicopathological characteristics of the 72 included patients are listed in Table 1. These patients included 41 men (56.9%) and 31 women (43.1%), and had a median age of 63 years (range: 30–80 years). Thirty-five patients (48.6%) were never-smokers and 37 patients (51.4%) were smokers. Thirty-six patients (50%) had tumors with a diameter of >3 cm. Some of the tumors exhibited pleural invasion (n = 35, 48.6%), vascular invasion (n = 29, 40.3%), and lymphatic invasion (n = 43, 59.7%). The pathological stages were stage I (44.4%), stage II (26.4%), stage III (23.6%), and stage IV (5.6%).

**Table 1:** Clinicopathological characteristics of the lung adenocarcinomas (n = 72)

Variables	N (%)
**Sex**	
Male	41 (56.9%)
Female	31 (43.1%)
**Age**	
median (range)	63 (30-80)
**Smoking status**	
Never	35 (48.6%)
Ever	37 (51.4%)
**Tumor size**	
≤3cm	36 (50%)
>3cm	36 (50%)
**Pleural invasion**	
Absent	37 (51.4%)
Present	35 (48.6%)
**Vascular invasion**	
Absent	43 (59.7%)
Present	29 (40.3%)
**Lymphatic invasion**	
Absent	29 (40.3%)
Present	43 (59.7%)
**Pathologic stage**	
I	32 (44.4%)
II	19 (26.4%)
III	17 (23.6%)
IV	4 (5.6%)
**Genetic status**	
*ALK*- rearranged	16 (22.2%)
*EGFR*-mutated	19 (26.4%)
*KRAS*-mutated	17 (23.6%)
Triple negative	20 (27.8%)

### Expression profiles of the 23 microRNAs and their associations with the clinicopathological variables

Among the 23 candidate microRNAs, expressions of 8 microRNAs were significantly associated with clinicopathological variables (Table 2). Compared to women and never-smokers, men and ever-smokers exhibited higher levels of miR-373-3p, miR-1343-3p, miR-138-1-3p, and miR-764 expression. Women and never-smokers exhibited higher levels of miR-27b-3p expression. Moreover, miR-27b-3p expression was associated with vascular invasion (*p* = 0.007), while miR-1343-3p and miR-764 expressions were inversely associated with vascular invasion (*p* = 0.047 and *p* = 0.028, respectively). Lymphatic invasion was marginally associated with low miR-342-3p expression (*p* = 0.052). Age, tumor size, pleural invasion, and pathological stage were not significantly associated with the microRNA expression levels.

**Table 2:** Association between representative microRNAs and the clinicopathological characteristics of lung adenocarcinomas (n = 72)

	miR-373-3p			miR-1343-3p				miR-671-3p				miR-937-3p				miR-138-1-3p				miR-647				miR-764	
	**mean± SD**	***p*** **value**		**mean± SD**		***p*** **value**		**mean± SD**		***p*** **value**		**mean± SD**		***p*** **value**		**mean± SD**		***p*** **value**		**mean± SD**		***p*** **value**		**mean± SD**	***p*** **value**
**Sex**																									
male	0.4±2.6	0.007*		0.3±1.2		0.045*		−1.1±1.7		0.027*		2.4±1.1		0.026*		−0.6±1.0		0.01*		−1.1±1.5		0.45		1.4±1.0	0.039*
female	−1.2±2.2			−0.2±0.9				−0.1±2.0				1.8±09				−1.2±1.2				−1.4±1.7				1.0±0.8	
**Age**																									
<60	−0.7±3.4	0.272		−0.2±0.1		0.062		−0.2±2.2		0.136		2.0±1.1		0.473		−1.1±1.1		0.235		−1.1±1.4		0.827		1.1±1.0	0.298
≥60	−0.1±2.0			0.3±1.1				−0.9±1.6				2.2±1.0				−0.7±1.1				−1.2±1.7				1.3±0.9	
**Smoking status**																									
never	−0.9±2.2	0.037*		−0.3±1.0		0.001*		−0.3±2.0		0.056		2.0±1.0		0.170		−1.2±1.2		0.026*		−1.2±1.6		0.836		0.9±0.6	0.007*
ever	0.3±2.7			0.5±1.0				−1.1±1.7				2.3±1.1				−0.6±1.0				−1.2±1.5				1.5±1.0	
**Tumor size**																									
≤3cm	−0.7±2.7	0.134		−0.1±1.2		0.130		−0.5±2.3		0.343		2.2±1.1		0.286		−0.8±1.3		0.762		−0.9±1.4		0.156		1.3±0.9	0.639
>3cm	0.2±2.3			0.3±0.9				−0.9±1.3				2.0±1.0				−0.9±1.0				−1.5±1.7				1.2±1.0	
**Pleural invasion**																									
absent	−0.2±2.2	0.753		−0.1±1.2		0.343		−0.9±1.8		0.383		2.0±1.1		0.415		−0.9±1.0		0.599		−1.1±1.6		0.740		1.4±0.9	0.216
present	−0.4±2.9			0.210.9				−0.5±2.0				2.2±1.0				−0.8±1.2				−1.3±1.6				1.1±0.9	
**Vascular invasion**																									
absent	0.1±2.7	0.133		0.3±1.0		0.047*		−0.9±2.0		0.258		2.2±1.1		0.419		−0.8±1.2		0.509		−1.4±1.6		0.282		1.4±1.0	0.028*
present	−0.8±2.2			−0.2±1.1				−0.4±1.7				2.0±1.0				−1.0±1.0				−1.0±1.5				0.9±0.7	
**Lymphatic invasion**																									
absent	0.1±2.9	0.424		−0.1±0.8		0.169		−0.8±2.0		0.730		1.9±1.2		0.233		−1.1±1.2		0.179		−1.3±1.8		0.827		1.4±1.1	0.316
present	−0.5±2.3			0.2±1.2				−0.6±1.8				2.2±1.0				−0.7±1.1				−1.2±1.5				1.1±0.8	
**Pathologic stage**																									
I	−0.6±2.3	>0.05		−0.3±1.1		>0.05		−0.5±2.0		>0.05		1.9±1.1		>0.05		−1.1±1.4		>0.05		−1.1±1.4		>0.05		1.3±0.9	>0.05
II	−0.3±1.8			0.4±0.8				−0.9±1.7				2.2±0.9				−0.7±0.7				−1.3±1.5				1.2±1.1	
III	0.7±3.4			0.5±1.0				−1.0±1.6				2.4±1.1				−0.6±0.7				−1.2±1.6				1.2±0.8	
IV	−1.2±3.3			−0.3±1.2				0.1±2.3				2.1±1.3				−0.8±1.8				−1.5±2.7				1.0±0.7	

	miR-200b-5p		miR-103a-3p				let-7e				miR-342-3p				miR-23b-3p				miR-27b-3p			
	**mean± SD**	***p*** **value**	**mean± SD**		***p*** **value**		**mean± SD**		***p*** **value**		**mean± SD**		***p*** **value**		**mean± SD**		***p*** **value**		**mean± SD**		***p*** **value**	
**Sex**																						
Male	3.2±1.7	0.401	−0.2±0.6		0.123		−0.1±0.7		0.123		−1.5±1.3		0.188		0.6±1.0		0.09		−1.5±1.3		<0.01*	
Female	2.8±1.4		0.1±0.5				0.2±1.0				−1.1±1.0				0.3±0.6				−0.5±0.7			
**Age**																						
<60	3.0±1.4	0.984	−0.5±0.6		0.771		0.0±0.9		0.831		−0.4±1.1		0.838		0.4±0.8		0.883		−0.7±1.1		0.079	
≥60	3.0±1.6		−1.0±0.6				0.1±0.9				−1.3±1.3				0.5±0.9				−1.2±1.2			
**Smoking status**																						
Never	2.8±1.5	0.264	0.5±0.5		0.059		0.3±0.9		0.029		−1.2±1.2		0.525		0.4±0.7		0.416		−0.4±0.7		<0.01*	
Ever	3.2±1.6		−0.2±0.6				−0.1±0.8				−1.4±1.3				0.5±1.0				−1.6±1.3			
**Tumor size**																						
≤3cm	3.0±1.6	0.964	0.1±0.5		0.115		0.1±0.9		0.686		−1.3±1.2		0.917		0.5±0.8		0.612		−0.9±1.2		0.416	
>3cm	3.0±1.5		−0.2±0.6				0.0±0.8				−1.3±1.3				0.4±0.9				−1.2±1.2			
**Pleural invasion**																						
Absent	3.0±1.6	0.977	−0.1±0.6		0.648		0.0±1.0		0.327		−1.4±1.4		0.764		0.3±0.9		0.245		−1.0±1.1		0.543	
Present	3.0±1.5		−0.1±0.6				0.2±0.7				−1.3±1.0				0.6±0.8				−1.1±1.3			
**Vascular invasion**																						
Absent	3.2±1.6	0.340	−0.1±0.6		0.305		0.0±0.9		0.190		−1.3±1.2		0.952		0.4±0.9		0.948		−1.3±1.3		0.007*	
Present	2.8±1.4		0.0±0.6				0.2±0.8				−1.3±1.2				0.5±0.8				−0.6±0.9			
**Lymphatic invasion**																						
Absent	2.7±1.9	0.093	−0.1±0.6		0.604		0.2±1.0		0.279		−1.0±1.3		0.052		0.3±0.9		0.214		−1.2±1.4		0.292	
Present	3.3±1.2		−0.1±0.6				0.0±0.8				−1.5±1.1				0.6±0.8				−0.9±1.0			
**Pathologic stage**																						
I	2.7±1.7	>0.05	−0.1±0.6		>0.05		0.2±0.9.		>0.05		−1.0±1.2		>0.05		0.3±0.8		>0.05		−0.9±1.2		>0.05	
II	3.2±1.4		−0.1±0.7				0.0±0.8				−1.4±1.5				0.4±1.0				−1.3±1.3			
III	3.4±1.7		0.0±0.5				−0.2±0.7				−1.6±1.0				0.8±0.6				−0.9±1.0			
IV	3.3±0.6		−0.2±0.8				−0.1±0.8				−1.7±1.0				0.5±0.8				−1.2±1.3			

Abbreviations: miR, microRNA; SD, standard deviation

* Statistically significant (*p* < 0.05)

### Differential expression patterns according to the four genetic groups

The differential microRNA expression patterns of the four genetic groups (*ALK*-rearranged*, EGFR*-mutated, *KRAS*-mutated, and triple negative [TN]) are shown in Supplementary Figure S1. Thirteen of the 23 microRNAs exhibited different expressions in the four genetic groups: miR-373-3p, miR-1343-3p, miR-671-3p, miR-937-3p, miR-138-1-3p, miR-647, miR-764, miR-200b-5p, miR-103a-3p, let-7e, miR-342-3p, miR-23b-3p, and miR27b-3p. The scatter plots for these 13 microRNAs revealed that the let-7e and miR-342-3p expression patterns were elevated in the *KRAS*-mutated lung adenocarcinomas (*KRAS* group) and *EGFR*-mutated lung adenocarcinomas (*EGFR* group), and reduced in the *ALK*-rearranged lung adenocarcinomas (*ALK* group) and TN lung adenocarcinomas (TN group).

### Clustering and discriminant analyses according to the four genetic groups

**Figure 1:**
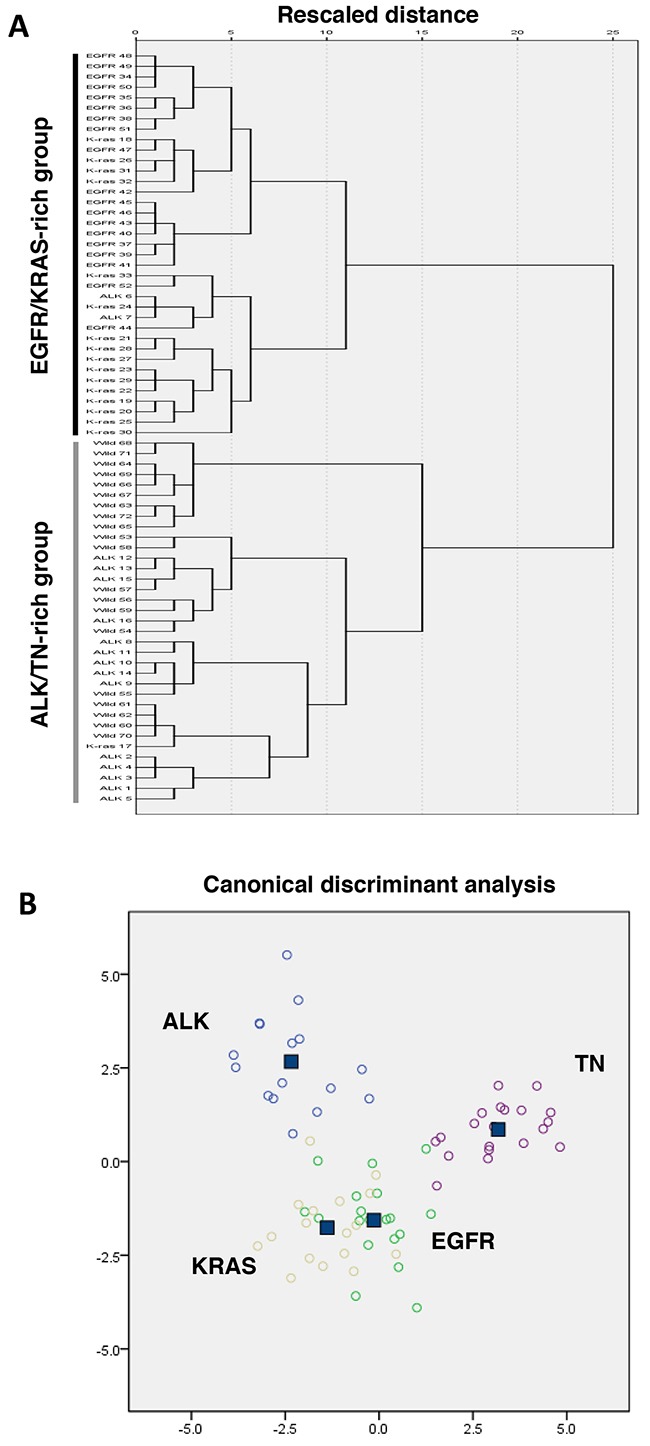
Clustering and discriminant analysis using the microRNAs expression profiles in lung adenocarcinomas **A.** A dendrogram using Ward's linkage and the expression profiles of 23 microRNAs revealed a trend towards two large groups: lung adenocarcinomas with EGFR or KRAS mutations (EGFR/KRAS-rich group) and lung adenocarcinomas with ALK rearrangement or triple-negative (TN) for these three genes (ALK/TN-rich group). **B.** Discriminant analysis of lung adenocarcinomas with EGFR and KRAS mutations and ALK rearrangement reveals relatively distinct grouping of the EGFR- and KRAS-mutated cancers, compared to the ALK-rearranged and triple negative cancers (wild type for EGFR, KRAS and ALK).

Hierarchical clustering analyses of the 23 microRNA expression levels revealed two large clusters: an *EGFR*/*KRAS*-rich group and an *ALK*/TN-rich group (Figure 1A). Discriminant analyses of the 23 microRNAs revealed that the *EGFR* and *KRAS* group were centered close to each other, and were relatively distant from the *ALK* and TN groups (Figure 1B). These findings suggest that the microRNA expression profiles were similar in the *EGFR* and *KRAS* groups, and that these groups had relatively distinct microRNA expression profiles, compared to the *ALK* and TN groups.

### Distinctive expressions of microRNAs in the ALK group and the EGFR/KRAS groups

**Figure 2:**
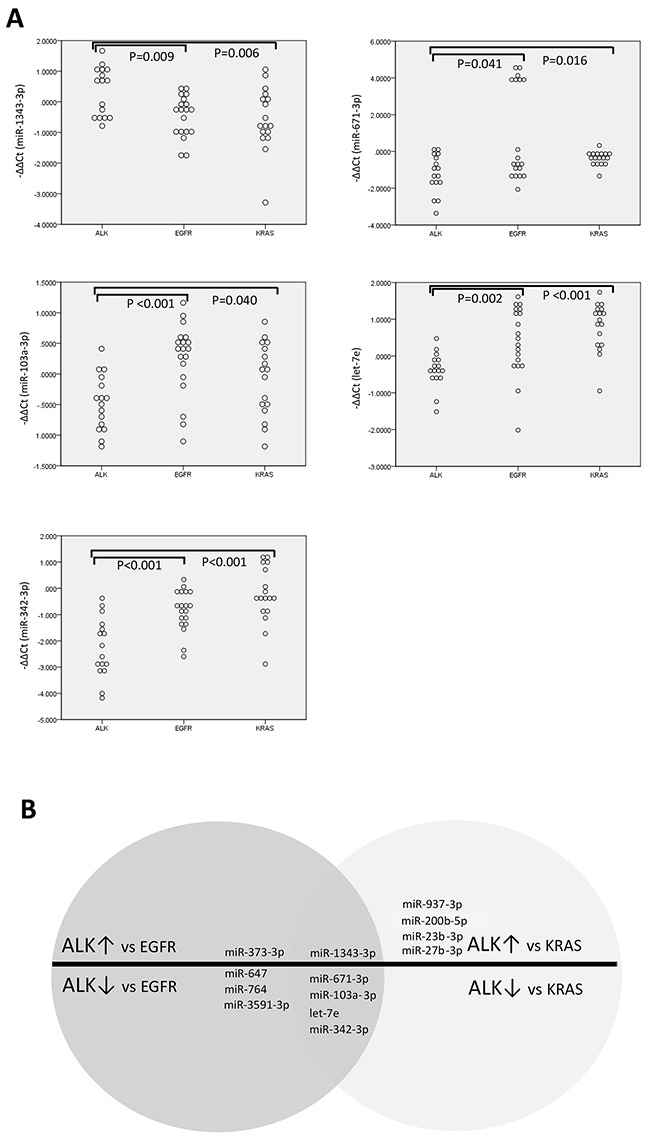
Dot plots of microRNA expressions in lung adenocarcinomas according to genetic alterations Dot plots **A.** and schematic illustration **B.** reveal differentially expressed microRNAs in ALK-rearranged cancers, compared to EGFR- or KRAS-mutated cancers. (n=52).

Based on the assumption that the TN group was composed of genetically heterogeneous cases, and the observation that the microRNA expression patterns were similar in the *EGFR* and *KRAS* groups, we focused on comparing the *ALK* group to the *EGFR* and *KRAS* groups. In the analyses of the *EGFR*, *KRAS*, and *ALK* groups (n = 52), the *ALK* group exhibited distinctive expression of five microRNAs (miR-1343-3p, miR-671-3p, miR-103a-3p, let-7e, and miR-342-3p), compared to the *EGFR* and *KRAS* groups (Figure 2). The *ALK* group had higher miR-1343-3p expression levels and decreased levels of miR-671-3p, miR-103a-3p, let-7e, and miR-342-3p expression.

The *EGFR* group was centered close to the *KRAS* group in the clustering and discriminant analyses. However, compared to the *KRAS* group, the *EGFR* group exhibited increased expression of miR-647, miR-200b-5p, miR-361-5p, miR23b-3p, and miR-27b-3p, with decreased expression of miR-23a-3p (Supplementary Figure S2).

### Association between microRNA and E-cadherin protein expressions in the ALK group and the EGFR/KRAS groups

**Figure 3:**
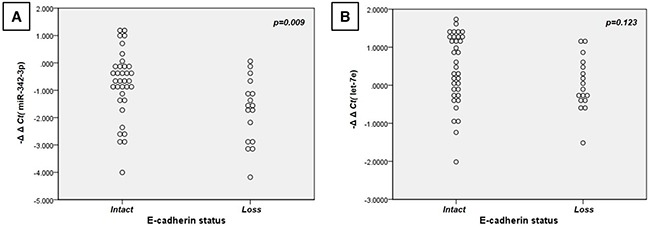
Dot-plots of miR -342-3p and let-7e expressions according to E-cadherin expression status miR-342-3p **A.** and let-7e **B.** exhibited lower expressions in patients with loss of E-cadherin in the ALK-rearranged, EGFR-mutated, and KRAS-mutated cancers (n = 52).

**Figure 4:**
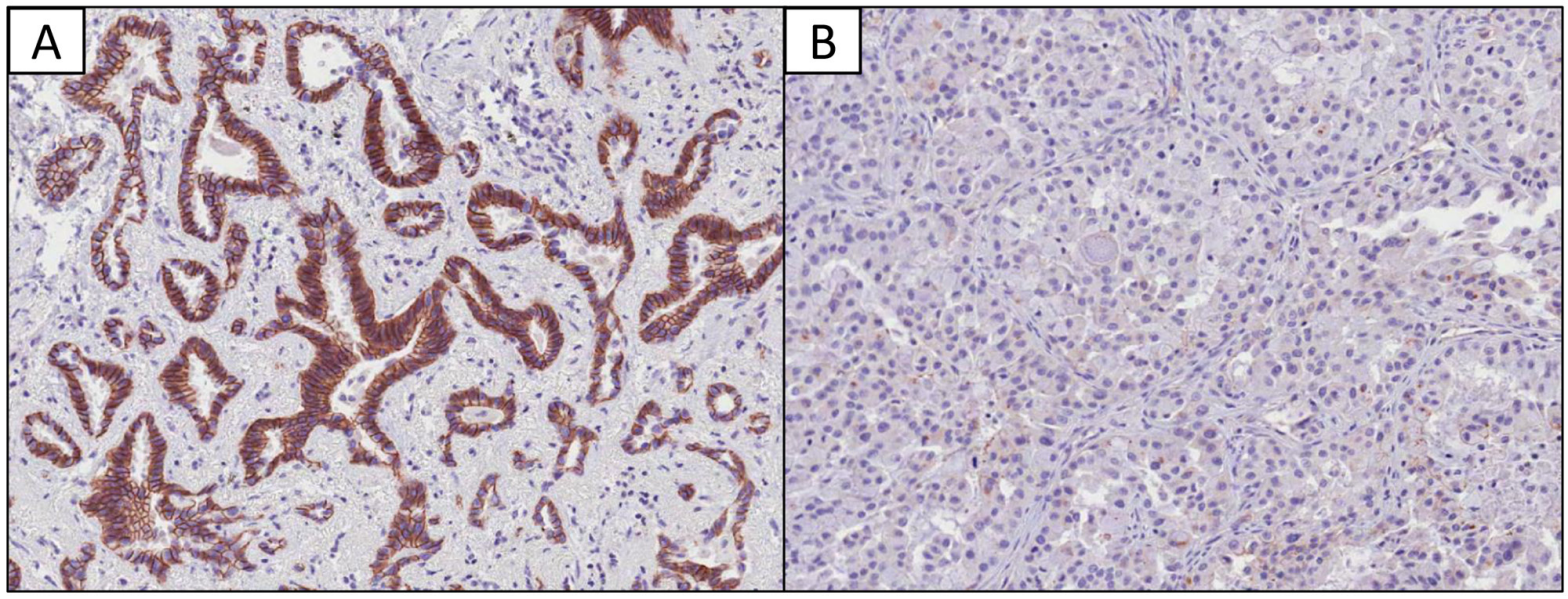
Immunohistochemical findings regarding E-cadherin expression in lung adenocarcinomas **A.** Normal membranous expression of E-cadherin (×20 magnification). **B.** Loss of membranous expression of E-cadherin (×20 magnification).

To investigate how *ALK*-distinctive microRNAs could contribute to the clinicopathological features of the *ALK* group, we focused on the frequent epithelial-mesenchymal transition (EMT) phenomenon in the *ALK* group and its relationship with the *ALK*-distinctive microRNAs [32]. For these analyses, we immunohistochemically evaluated the loss of E-cadherin expression (a well-known EMT marker) and two *ALK*-distinctive microRNAs (let-7e and miR-342-3p), which are associated to EMT and cancer cell migration and invasion [33, 34]. As shown in Table 3 and Supplementary Table 1, the *ALK* group exhibited low levels of let-7e and miR-342-3p expression, and loss of E-cadherin expression. The expressions of miR-342-3p, let-7e and E-cadherin tended to be closely related (Figure 3). Figure 4 shows the immunohistological staining results for E-cadherin. These findings indicate that the *ALK* group could be characterized by decreased miR-342-3p and let-7e expression and frequent loss of E-cadherin, which suggests increased EMT.

**Table 3:** Correlations of *ALK* rearrangement, miR-342-3p levels, let-7e levels, and E-cadherin expression in the lung adenocarcinomas of the *EGFR*, *KRAS*, and *ALK* groups (n = 52)

	miR-342-3p level		let-7e level		E-cadherin expression	
	***ρ***	***p*** **value**	***ρ***	***p*** **value**	***ρ***	***p*** **value**
***ALK*** **rearrangement**	−0.616	*p*<0.001*	−0.525	*p*<0.001*	−0.424	*p*=0.002*
**miR-342-3p level**			+0.537	*p*<0.001*	+0.359	*p*=0.009*
**let-7e level**					+0.217	*p*=0.123

* Statistically significant (*p* < 0.05)

## DISCUSSION

Lung adenocarcinoma represents a heterogeneous group of tumors with broad spectrums of morphology, clinical behavior, genetic alteration, and therapeutic response. Furthermore, the discovery of driver oncogenes (e.g., *EGFR*, *KRAS* and *ALK*) has changed our understanding of and approach to treating lung carcinogenesis, which has highlighted the importance of the genotype in lung cancer research [35, 36]. Thus, we aimed to provide insight into lung adenocarcinoma biology according to various genotypes, and investigated whether microRNA expression profiles could be used to characterize the genotypic groups of lung adenocarcinomas. Several reports demonstrated that unique miRNA expressions were related with genotypes in lung adenocarcinoma [37–39]. Although a few miRNAs such as miR-184 was also identified in our study, it was difficult to compare or integrate the results due to the differences in study design and methods between these studies and ours. In addition, we compared with four different genotypes in lung adenocarcinoma, which has not yet been performed in other studies. The present study revealed that 1) several microRNAs’ expression levels were associated with clinicopathological features of lung cancers (e.g., miR-27b-3p expression was closely associated with vascular invasion), 2) the microRNA expression profiles in *ALK*-rearranged cancers were different from those in *EGFR/KRAS*-mutated cancers, 3) *ALK*-rearranged cancers exhibited characteristic expression changes for five microRNAs (including let-7e and miR-342-3p), compared to *EGFR/KRAS*-mutated cancers, and 4) the loss of E-cadherin expression was associated with *ALK*-rearrangement and decreased miR-342-3p expression, which suggests that miR-342-3p may play a role in the EMT of *ALK*-rearranged cancers.

MicroRNAs are precise regulators of various physiological processes and disease models, such as cancers and vascular diseases [11, 12, 14, 15, 40, 41]. In addition, vascular invasion is an important step in metastasis to distant sites, and angiogenesis and its regulators are associated with tumor growth and prognosis in various solid tumors, including lung cancer [42, 43]. In this context, miR-27b-3p is a pro-angiogenic microRNA [40, 44] that is up-regulated in vascular endothelial cells [45–47]. In a recent mouse study, increased miR-27b-3p expression resulted in enhanced neovascularization and contributed to tumor growth [44]. These findings may help explain the association between increased miR-27b-3p and vascular invasion in our study. However, given our limited ability to measure microRNA expressions in the complete specimens (intermixed tumor, vascular, stromal, and other microenvironmental cells), the precise source of the increased miR-27b-3p expression in lung cancer with vascular invasion remains unclear. For example, pro-angiogenic miR-27b-3p may be actively produced and secreted by tumor cells to permeabilize the vessels and facilitate vascular supply and tumor growth, or there may be secondary production as part of a vascular regenerative process after invasion by the tumor cells. Further studies are needed to evaluate this issue. Other reports have also revealed that miR-1343-3p reduces the expression of transforming growth factor-β (TGF-β) receptor-1, which induces angiogenesis through vascular endothelial growth factor (VEGF)-mediated apoptosis [48, 49]. Therefore, miR-1343-3p may also play an anti-angiogenic role that could oppose the pro-angiogenic role of miR-27b-3p. Moreover, little is known regarding about the angiogenic effects of miR-764. Databases of microRNAs (e.g.,microrna.org) suggest that RAF-1 may be an important target of miR-764, and may also promote angiogenesis [50, 51]. Thus, miR-764 may mediate angiogenesis through RAF-1, although further studies are also needed to evaluate this possibility. Moreover, our data indicate that miR-1343-3p and miR-764 expressions were inversely associated with vascular invasion and may play anti-angiogenic roles, and while miR-27b-3p expression was positively associated with vascular invasion and may play a pro-angiogenic role.

MiR-27b-3p and miR-23a/b are functionally co-operating microRNA clusters that play roles in preventing apoptosis, promoting angiogenesis, and promoting metastasis [52–55]. Interestingly, we observed that low miR-23a and high miR-23b-3p/27b-3p expression profiles were characteristic of our *EGFR*-mutated cancers, but were not characteristics of the *KRAS*-mutated lung cancers. Thus, these expression profiles may affect the biology and molecular features of *EGFR*-driven tumors, in addition to their associations with various clinicopathological features (female sex, non-smoker status, Asian descent, and old age) [4, 56–58].

*ALK*-rearranged cancers have distinct and aggressive clinicopathological characteristics, such as a young age at presentation, frequent nodal metastasis, high disease stage at the diagnosis, cribriform formation, presence of mucin-containing cells, presence of psammoma bodies, predominantly solid patterns, and frequent EMT [32]. In our previous study, *ALK-*rearranged tumors were closely related to the adjacent bronchial epithelium, especially the proximal bronchus, and tended to be centrally located [32]. These distinctive features of *ALK*-rearranged cancers may be the result of pathologically activated *ALK* signaling, although the detailed underlying molecular mechanisms remain unclear. In addition, activated *ALK* signaling can influence the expressions of several important protein-coding genes that are involved in the JAK/STAT, PI3K/AKT and RAS/ERK pathways [59], as well as non-coding RNAs, such as miR-150 and miR-155 [60, 61]. Given the complex roles of microRNAs as “fine tuners” of gene expression [62], it is conceivable that the microRNA expression profile of *ALK*-rearranged cancers might be at least partially different from *EGFR*- and *KRAS*-driven cancers, which partly share signaling pathways. Consistent with this hypothesis, our findings revealed that *ALK*-rearranged cancer had distinctive microRNA expression profiles, while the *EGFR*- and *KRAS*-mutated cancers had similar microRNA expression profiles, despite having several differentially expressed microRNAs. It is possible that commonly activated RAS/MAPK signaling pathways in these groups could largely influence their microRNA expression profiles, although further clarification is needed.

Another remarkable finding was the distinctive low expressions of miR-342-3p and let-7e in *ALK*-rearranged cancer, compared to *EGFR/KRAS*-mutated cancers. A previous study of a pancreatic cancer cell line revealed that the expression of the let-7 family (including let-7e) was decreased in gemcitabine-resistant cells with EMT features, such as fibroblastoid morphology and low E-cadherin expression [33]. Another study of the A549 lung cancer cell line revealed reduced let-7 levels in side population cells (an enriched source of cancer stem cells), compared to non-side population cells [63]. These findings suggest that let-7e down-regulation might be associated with stemness, chemoresistance, and EMT, which might contribute to the potentially aggressive tumor biology of *ALK*-rearranged lung cancer. Interestingly, a recent study suggested that the RAS-mitogen-activated protein kinase (MAPK) pathway was an important mechanism of resistance to ALK inhibitors in *ALK*-rearranged lung cancer [64]. As let-7e and the let-7 family are regulators of RAS [28, 65], it is possible that RAS activation is mediated by low let-7e levels. This provides an hypothetical mechanism whereby the RAS-MAPK axis is activated in *ALK*-rearranged tumor cells and allows them to resist suppression by ALK inhibitors.

*ALK*-rearranged lung cancer was also associated with low miR-342-3p expression and E-cadherin loss. In this context, E-cadherin is a cell-cell adhesion molecule and its loss is an important marker for the EMT process that allows cancer cells of epithelial origin to gain migratory and invasive properties [66]. Furthermore, the significant association between *ALK* rearrangement, low miR-342-3p expression, and E-cadherin loss might contribute to the unique clinicopathological features of *ALK*-rearranged lung cancer [32]. Several reports have suggested that miR-342-3p expression might be related to E-cadherin through DNMT1, which is a target mRNA for miR-342-3p, based on miRNA databases (e.g.,microrna.org). Although DNMT1 is an essential DNA methyltransferase, it might affect E-cadherin expression through its direct and DNA methylation-independent interaction with SNAIL (an E-cadherin transcriptional repressor) [67]. In non-small cell lung cancer, miR-342-3p has been suggested to play a tumor-suppressive role by negatively regulating RAP2B (a member of the RAS oncogene family) [68] and MYC transcriptional activity by targeting E2F1 (a MYC-cooperating molecule) [69]. In this context, the simultaneous down-regulation of miR-342-3p and let-7e in *ALK*-rearranged lung cancer might help amplify the RAS family effector signals and/or MYC activity, which could result in resistance to anti-cancer drugs [64, 70]. Therefore, it might be useful to investigate miR-342-3p and let-7e mimics in combination with *ALK* inhibitors for *ALK*-rearranged lung cancers, in order to prevent drug resistance and EMT-mediated metastasis. However, this approach requires further support from functional validation studies, pre-clinical studies, and clinical trials.

In conclusion, our results revealed that microRNA expression profiles had clinicopathological implications that were related to *EGFR* and *KRAS* mutations, as well as *ALK*-rearrangement. *ALK*-rearranged lung cancer had a unique microRNA expression profile that was distinct from that of *EGFR-* and *KRAS*-mutated lung cancers. In addition, *ALK*-rearranged lung cancer was associated with decreased miR-342-3p and let-7e expression, and the loss of E-cadherin. These characteristics may provide insight regarding the mechanisms for the relatively aggressive biology of *ALK*-rearranged lung cancer, as well as potential therapeutic strategies to overcome resistance to ALK inhibitors and prevent EMT-mediated metastasis.

## MATERIALS AND METHODS

### Patients and samples

We retrieved 72 lung adenocarcinoma paraffin-embedded specimens that had been surgically resected at Seoul National University Bundang Hospital between January 2004 and June 2011. Among these specimens, 19 were *EGFR*-mutated, 17 were *KRAS*-mutated, 16 were *ALK*-rearranged, and 20 were TN (negative for *EGFR* mutations, *KRAS* mutations, and *ALK* rearrangement). Hematoxylin and eosin-stained slides of all specimens were reviewed by two pulmonary pathologists (HK and JHC) and confirmed to be adenocarcinoma. All cases were classified according to the seventh edition of the Union for International Cancer Control/American Joint Committee on Cancer TNM classification [71]. Clinicopathological data were obtained from the patients’ medical records and pathology reports. This study's retrospective design was approved by the institutional review board at Seoul National University Bundang Hospital.

### Detection of EGFR and KRAS mutations

Genomic DNA was extracted from paraffin-embedded tissues. After deparaffinization using xylene, tissue sections were stained using hematoxylin and eosin, and the target lesions were selectively dissected to minimize contamination with normal tissue. Genomic DNA was isolated using the QIAamp DNA Mini Kit (Qiagen, Hilden, Germany) according to the manufacturer's instructions. *EGFR* mutations at exons 18–21 and *KRAS* mutations at codons 12, 13, and 61 were analyzed using nested polymerase chain reaction (PCR) and direct DNA sequencing, as previously described [72]. PCR products were processed using the Big Dye Terminator v3.1 Cycle Sequencing Kit (Applied Biosystems, Foster, CA, USA), and the sequence data were generated using the ABI PRISM 3100 DNA Analyzer (Applied Biosystems).

### Detection of ALK gene rearrangement

*ALK* rearrangement in formalin-fixed paraffin-embedded (FFPE) tumor tissues was detected using fluorescence *in situ* hybridization and a break-apart probe that is specific for the *ALK* locus (Vysis LSI *ALK* dual-color, break-apart rearrangement probe; Abbott Molecular, Abbott Park, IL, USA). Positive cases were defined as cases with >15% split signals or an isolated red signal in the tumor cells, as previously described [73–75].

### RNA extraction from FFPE tissue samples

Total RNA was extracted as previously described [76]. The RecoverAll Total Nucleic Acid Isolation Kit (Applied Biosystems) was used to isolate RNA from 10 mm-thick FFPE tissue sections. The concentrations were measured using a NanoDrop 2000 spectrophotometer (Thermo Fisher Scientific Inc., Waltham, MA, USA), and then the RNAs were stored at 80°C until the time of use.

### Screening and selection of microRNAs for quantitative analysis

To quantitatively evaluate the microRNA expressions, the GeneChip® miRNA 3.0 Array (Affymetrix, Santa Clara, CA, USA) was used to compare four cancer RNA samples (*EGFR*, *KRAS*, *ALK*, and TN) to one control RNA sample. Each of the five RNA samples was extracted from 10 mixed FFPE cancer tissues (*EGFR*, *KRAS*, *ALK*, and TN) or non-neoplastic lung tissue. Twenty-three microRNAs were selected from a candidate pool of microRNAs that were identified using GeneChip screening, which was based on: 1) the precise expression levels (>6-fold) in at least one genetic group (vs. the other genetic groups) or 2) the precise expression levels (>10-fold) in at least one genetic group (vs. non-neoplastic lung tissue). The selected microRNAs were miR-373-3p, miR-3591-3p, miR-3160-5p, miR-4254, miR-1343-3p, miR-671-3p, miR-937-3p, miR-212-3p, miR-138-1-3p, miR-647, miR-764, miR-184, miR-200b-5p, miR-361-5p, miR-23a-3p, miR-103a-3p, let-7e, miR-342-3p, miR-23b-3p, miR27b-3p, miR-4513, miR-205-5p, and miR-31-5p.

### Quantitative real-time PCR analysis of microRNA expression

The reverse transcription and real-time PCR was performed as previously described [76]. For each sample, and according to the manufacturer's instructions, 10 ng of total RNA were mixed with the TaqMan MicroRNA Reverse Transcription Kit (Applied Biosystems) and the RT primers that were included in the TaqMan MicroRNA Assay (Applied Biosystems; catalogue numbers: 000561 (miR-373-3p), 464780_mat (miR-3591-3p), 464424_mat (miR-3160-5p), 244545_mat (miR-4254), 463957_mat (miR-1343-3p), 002322 (miR-671-3p), 002180 (miR-937-3p), 000515 (miR-212-3p), 002162 (miR-138-1-3p), 001600 (miR-647), 241115_mat (miR-764), 000485 (miR-184), 002274 (miR-200b-5p), 000554 (miR-361-5p), 000399 (miR-23a-3p), 000439 (miR-103a-3p), 002406 (let-7e), 002260 (miR-342-3p), 000400 (miR-23b-3p), 000409 (miR-27b-3p), 464329_mat (miR-4513), 000509 (miR-205-5p), 001100 (miR-31-5p), and 001973 (U6 snRNA)). We also used the StepOnePlus Real-Time System (Applied Biosystems) and related products. The signal from the FAM dye (490 nm) was collected during 50 cycles of amplification, and the threshold cycle (Ct) value was normalized using U6 snRNA as reference RNA: ΔCt (miR, cancer) = Ct (miR, cancer) – Ct (U6, cancer). The value was also adjusted using the expression of microRNA in non-neoplastic lung tissue (ΔCt = miR, normal). Thus, the relative expressions of microRNA in the cancer specimens were calculated as –ΔΔCt = – [ΔCt (miR, cancer) – ΔCt (miR, normal)].

### Construction of tissue microarrays

The tissue microarray blocks were constructed by Superbiochips Laboratories (Seoul, Korea) using the most representative areas of the paraffin blocks, as previously described [77].

### Immunohistochemical analysis of E-cadherin expression

Immunohistochemistry was performed using tissue microarray sections. Four-micrometer-thick sections were transferred to poly-l-lysine-coated glass slides and incubated in a dry oven at 60°C for 1 h. The sections were then dewaxed using xylene (3 changes), rehydrated in a descending series of graded ethanol concentrations, and rinsed using Tris-buffered saline (pH 7.4). The endogenous peroxidase activity was blocked using 5% hydrogen peroxide in methanol for 15 min at 37°C. For antigen retrieval, the slides were placed in citrate buffer (10% citrate buffer stock in distilled water, pH 6.0) and microwaved for 10 min. Non-specific staining was blocked using 1% horse serum in Tris-buffered saline (pH 7.4) for 3 min. The primary antibody for E-cadherin was diluted 1:100 (BD Biosciences, San Jose, CA), and the immunostaining was developed using an avidin-biotin-peroxidase complex (Universal Elite ABC Kit; PK-6200; Vectastain, Burlingame, CA, USA) and diaminobenzidine tetrahydrochloride solution (HK153-5K; Biogenex, San Ramon, CA, USA). Positive controls (samples with known reactivity for the antibody) and negative controls (omission of the primary antibody) were included in each assay. Positive E-cadherin staining was defined as a cytoplasmic membranous pattern, and was classified as high (>90%), low (0–90%), or absent (0%) [78].

### Statistical analysis

All data analyses were performed using SPSS software (version 21.0; SPSS Inc., Chicago, IL, USA). The associations between the clinicopathological parameters and the microRNA expression patters were evaluated using Pearson's R value, the chi-square test, and Fisher's exact test. The Mann-Whitney U test and Kruskal-Wallis test were used to compare the microRNA expression levels between the genetic groups. Hierarchical clustering analyses were performed using Ward's method, with intervals measured using the square Euclidian distance and standardized values (0 to 1). All tests were two-tailed, and differences with *p*-values of <0.05 were considered statistically significant.

## SUPPLEMENTARY MATERIALS FIGURES AND TABLES


