# Supplementary material for: MicroRNA expression profiles and clinicopathological implications in lung adenocarcinoma according to EGFR, KRAS, and ALK status
Source: Oncotarget. 2016 Dec 27;8(5):8484–98. doi: 10.18632/oncotarget.14298 (PMC5352416; doi:10.18632/oncotarget.14298)
Supplement: Supplementary file 1 [file oncotarget-08-8484-s001.pdf]

# MicroRNA expression profiles and clinicopathological implications in lung adenocarcinoma according to *EGFR*, *KRAS*, and *ALK* status

## SUPPLEMENTARY MATERIALS

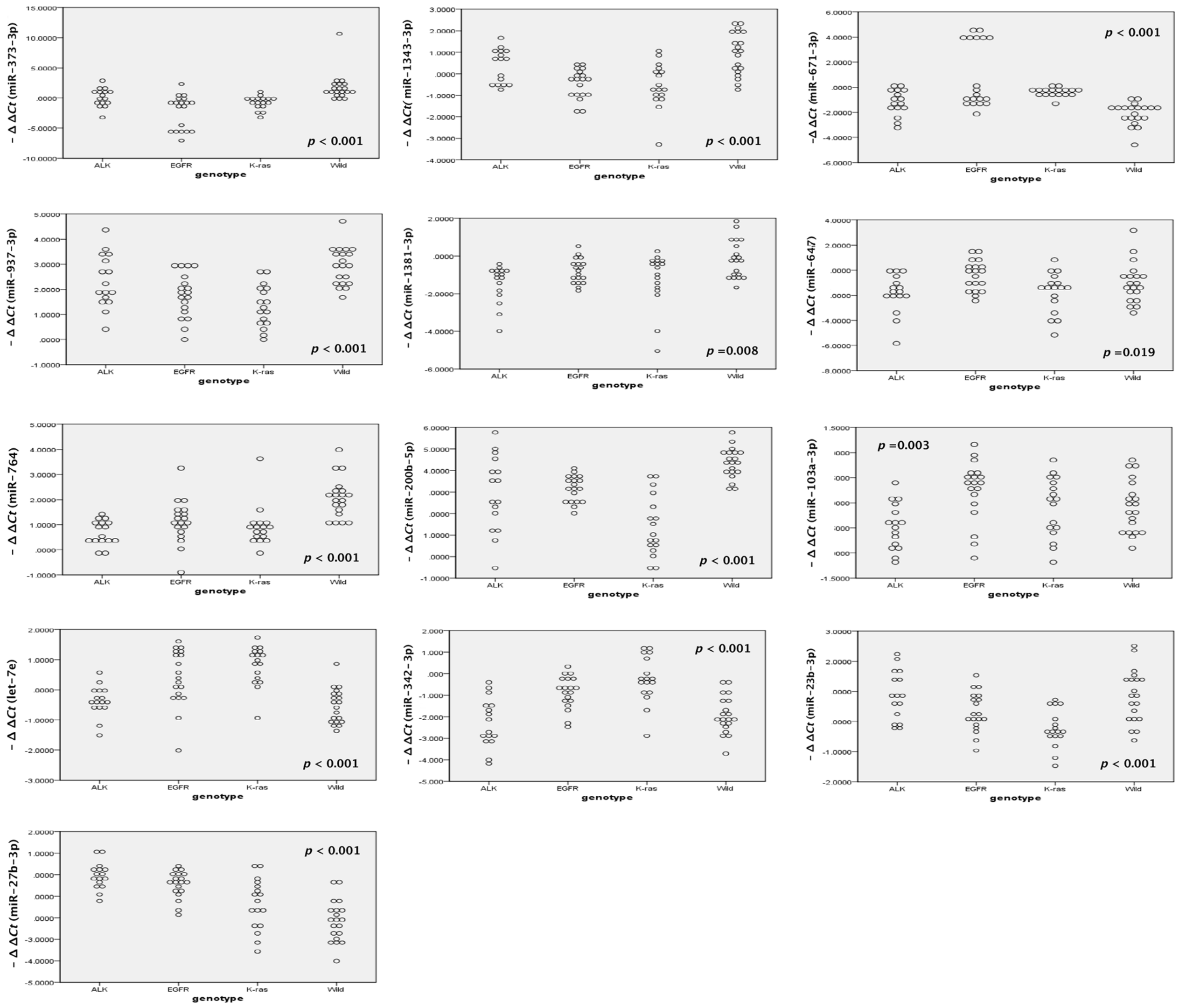


**Supplementary Figure 1: Dot plots of microRNA expressions in lung adenocarcinomas according to genetic status (*ALK*-rearranged, *EGFR*-mutated, *KRAS*-mutated, and triple negative).**

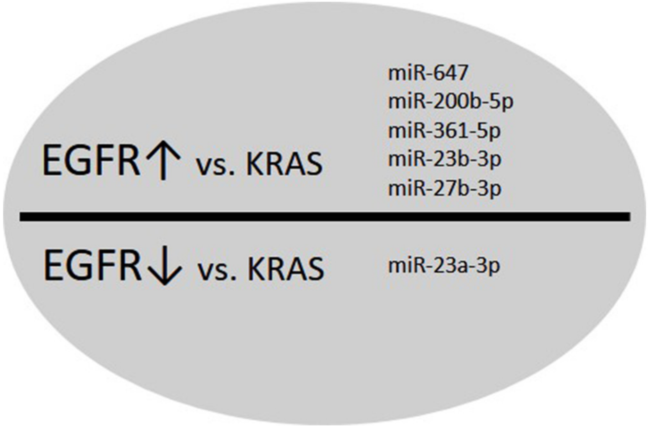


**Supplementary Figure 2: Differential expressions of microRNAs in *EGFR*-mutated lung adenocarcinomas, compared to *KRAS*-mutated lung adenocarcinomas.**

**Supplemetary Table 1: Association between *ALK* rearrangement and E-cadherin expression in lung adenocarcinomas of *EGFR*, *KRAS* and *ALK* groups (n = 52)**

| | Total N(%) | E-cadherin, N(%) | | |
|---|---|---|---|---|
| | | No loss | Loss | *p* value |
| **ALK** | 16 (30.8) | 6 (37.5) | 10(62.5) | 0.002* |
| **EGFR+KRAS** | 36 (69.2) | 29(80.6) | 7 (19.4) | |

* Statistically significant ($p < 0.05$)
